# Anti-phage serum antibody responses and the outcome of phage therapy

**DOI:** 10.1007/s12223-020-00835-z

**Published:** 2020-10-30

**Authors:** M. Łusiak-Szelachowska, R. Międzybrodzki, W. Fortuna, J. Borysowski, Andrzej Górski

**Affiliations:** 1 Bacteriophage Laboratory, Hirszfeld Institute of Immunology and Experimental Therapy, Polish Academy of Sciences (HIIET PAS), Wrocław, Poland; 2 Phage Therapy Unit, Hirszfeld Institute of Immunology and Experimental Therapy, Polish Academy of Sciences (HIIET PAS), Wrocław, Poland; 3 Department of Clinical Immunology, Transplantation Institute, Medical University of Warsaw, Warsaw, Poland; 4 Department of Neurosurgery, Wrocław Medical University, Wrocław, Poland; 5 Infant Jesus Hospital, Medical University of Warsaw, Warsaw, Poland

## Abstract

We examined the appearance of serum anti-phage antibodies in 25 patients with chronic sinusitis treated with phage therapy (PT). Approximately 30% of patients with weak antibody responses responded positively to PT, which was similar to the results of treatment achieved in a group of patients with high antibody production. In addition, there was no correlation between antibody level and the outcome of PT. These data, derived from a homogenous group of patients, confirm our earlier findings suggesting that the prognostic significance of serum anti-phage antibodies for the outcome of PT should be determined by relevant clinical trials.

## Introduction

The growing menace of antibiotic resistance has greatly revived interest in phage therapy (PT). In the past few years, a number of reports have been published strongly suggesting that the PT—alone or in association with antibiotics—may be an efficient means of combating multidrug-resistant bacteria. Some authors believe that antibiotic marketplace is broken and that the therapy with bacterial viruses has the potential to be used in the treatment of infectious diseases either alone or as adjuvants to existing therapies (Safir et al. 2020). A number of clinical trials are currently underway which suggests that the real value of PT should eventually be determined according to currently required standards of evidence-based medicine.

Phages—as other viruses—may interact with the immune system. Such interactions can lead to anti-phage antibody responses as well as cause immunomodulating effects. In the past years, phage-mediated immunomodulation has received increasing attention. Phage-dependent immunomodulating effects may have therapeutic potential in disorders of the immune system and alleviation of allograft reactivity as well as treatment of some non-bacterial infections (Górski et al. 2019a). Our studies have supplied some data on anti-phage humoral responses of mice receiving phages as well as phage proteins using different routes of phage administration and duration of phage treatment (Dąbrowska 2019). Furthermore, we studied anti-phage antibody formation in patients receiving PT (Łusiak-Szelachowska et al. 2014). The results of those studies suggest that humoral responses elicited by PT may depend on route of administration, phage type, duration of the therapy, and patient’s immunocompetence. Our initial observations also suggest that there is no apparent association of intensity of humoral anti-phage responses and therapy outcome (Górski et al. 2019b). This observation is somewhat unexpected, as it could be anticipated that such phage neutralizing antibodies would compromise the beneficial effects of phages in patients receiving PT. Evidently, the relationship of anti-phage antibodies and the success of therapy is of obvious practical significance for further advancement of PT, especially planning of decisive clinical trials. Interestingly, available reports of recent clinical trials completed so far do not provide any relevant information so it is impossible to determine how anti-phage antibodies could have contributed to trial outcome (Ujmajuridze et al. 2018; Jault et al. 2019; Ooi et al. 2019). In this report, we confirm and extend our earlier preliminary data (Łusiak-Szelachowska et al. 2017), but currently derived from a homogenous group of patients (one clinical model) suggesting that the appearance of serum anti-phage antibodies does not exclude positive outcome for PT.

## Materials and methods

### Patients

Twenty-five patients with chronic rhinosinusitis undergoing PT at our Phage Therapy Unit (PTU) under the therapeutic protocol “Experimental phage therapy of drug-resistant bacterial infections, including MRSA infections” (Międzybrodzki et al. 2012) were included. Patients used phage preparations locally (*n* = 4) or locally and orally (*n* = 21). Topical application of phages was accomplished by sinus irrigation (2 × 15 mL of phage) or nasal spraying. Topical and oral application was accomplished by nasal drops (2 × 4 mL of phage) and orally (2 × 6 mL of phage). Before oral phage administration, 10 mL of dihydroxyaluminum sodium carbonate (68 mg/mL) was applied orally. Sera were obtained from patients between 2010 and 2019. Sera of voluntary blood donors were obtained from the Blood Transfusion Center in Wrocław, Poland. All subjects gave written informed consent and the study was approved by the Bioethics Committee at Wrocław Medical University (Poland). The blood was centrifuged at 1500×*g* for 10 min and sera were stored at − 70 °C. Blood was collected before, during, and after PT. The neutralization test of phages by sera was performed immediately after obtaining sera. Sera were investigated during PT from day 14 to day 63. The highest *K* rate during PT was taken to analysis.

### Bacteriophage preparations

Patients used monovalent lysates of *Staphylococcus aureus*, *Pseudomonas aeruginosa*, *Klebsiella pneumoniae* or *Escherichia coli* phages (22 patients), *S. aureus* MS-1 phage cocktail (1 patient), and purified *S. aureus* OPMS-1 phage cocktail (1 patient) or purified *S. aureus* OPMS-1 top phage cocktail (1 patient) based on phage typing (Table 1; Table 2). Patients applied mainly *S. aureus* phage preparations (23 patients). *S. aureus* phage cocktails MS-1, OPMS-1, and OPMS-1 top are composed of three *S. aureus* phages—676/Z, A5/80, P4/6409. Purified phage cocktail OPMS-1 contained phages suspended in phosphate-buffered saline (PBS) with addition of saccharose (stability of storage conditions). Purified phage cocktail OPMS-1 top was deprived of saccharose. Phage preparations were used in the following concentrations: monovalent phage lysate 10^6^–10^8^ plaque forming unit/mL (pfu/mL), *S. aureus* MS-1 phage lysate cocktail 10^6^–10^8^ pfu/mL, and purified *S. aureus* OPMS-1 and OPMS-1 top phage cocktails 10^9^ pfu/mL.

**Table 1:** Patients with chronic rhinosinusitis with positive responses to PT

Patient	Phage preparation used in PT	Route of phage administration	Phage inactivation (*K*) before PT	Phage inactivation (*K*) during PT^a^	Day of PT on which *K* was marked	Clinical outcome of PT (A–C)^b^
1	Staph OPMS-1	Locally	0.74	28.28	62	C
2	Staph A5/80 Since 14th day of PT Staph 676/T	Locally	0.06 0.85	0.16 12.06	14 55	A
3	Pseud Psmw31	Locally and orally	0.00	0.002	14	B
4	Staph OPMS-1top	Locally and orally	0.25	4.25	15	C
5	Staph 676/T	Locally and orally	0.36	48.21	49	A
6	Staph 676/Z	Locally and orally	0.03	1.21	48	B
7	Klebs Kl 16/30 Coli 126/2031	Locally and orally	0.005 0.01	0.02 0.11	31	C
8	Staph A5/L	Locally and orally	0.03	0.72	14	C
			Mean *K* ± *SD* 0.23 ± 0.32	Mean *K* ± SD* 9.50 ± 16.28 (Wilcoxon test, *p* = 0.005)	14–62	

*K* rate of phage inactivation, *PT* phage therapy, *SD* standard deviation

*K* < 5, low neutralization of phages

*K* = 5–18, medium neutralization of phages

*K* > 18, high neutralization of phages

^a^Maximum *K* achieved during PT

^b^Results A–C positive responses to PT

*Significantly associated with the increase in the *K* rate during PT compared to the *K* rate before PT (Wilcoxon test; *p* = 0.005). There was no statistically significant difference (Mann-Whitney *U* test; *p* = 0.65) in the *K* rate during PT between positive responses to PT (Table 1) and inadequate responses to PT (Table 2)

**Table 2:** Patients with chronic rhinosinusitis with inadequate responses to PT

Patient	Phage preparation used in PT	Route of phage administration	Phage inactivation (*K*) before PT	Phage inactivation (*K*) during PT^a^	Day of PT on which *K* was marked	Clinical outcome of PT (D–G)^b^
1	Staph 676/Z	Locally	0.37	24	51	E
2	Staph ϕ200	Locally	0.03	0.12	14	F
3	Staph MS-1	Locally and orally	0.07	0.26	31	F
4	Staph P4	Locally and orally	0.21	0.24	21	E
5	Staph ϕ200	Locally and orally	0.33	0.21	26	E
6	Staph A5/L	Locally and orally	0.11	75.12	56	E
7	Staph 1 N/80	Locally and orally	0.02	1.42	17	E
8	Staph A5/L	Locally and orally	0.02	68.94	30	E
9	Staph 676/F	Locally and orally	0.00	0.9	32	F
10	Staph 676/F	Locally and orally	0.02	0.14	17	F
11	Staph 676/F	Locally and orally	0.03	0.07	63	F
12	Staph 676/T Coli 76/850	Locally and orally	0.35 0.00	0.42 0.007	14	G
13	Staph 676/T	Locally and orally	0.00	22.53	26	G
14	Staph 1 N/80	Locally and orally	0.34	0.53	47	F
15	Staph 1 N/80	Locally and orally	0.19	0.67	18	F
16	Staph 676/T	Locally and orally	0.52	0.92	18	F
17	Staph ϕ200	Locally and orally	0.00	0.15	14	F
			Mean *K* ± *SD* 0.14 ± 0.17	Mean *K* ± SD* 10.92 ± 23.43 (Wilcoxon test, *p* = 0.0006)	14–63	

*K* rate of phage inactivation, *PT* phage therapy, *SD* standard deviation

*K* < 5, low neutralization of phages

*K* = 5–18, medium neutralization of phages

*K* > 18, high neutralization of phages

^a^Maximum *K* achieved during PT

^b^Results D–G inadequate responses to PT

*Significantly associated with the increase in the *K* rate during PT compared to the *K* rate before PT (Wilcoxon test; *p* = 0.0006). There was no statistically significant difference (Mann-Whitney *U* test; *p* = 0.65) in the *K* rate during PT between positive responses to PT (Table 1) and inadequate responses to PT (Table 2)

### Phage neutralization test

Phage neutralization by human sera was performed as described earlier (Łusiak-Szelachowska et al. 2017). Anti-phage activity of sera (AAS) was calculated as phage inactivation (*K* rate), where *K* less than 5 was considered as a low neutralization of phages, *K* between 5 and 18 as a medium level, and above 18 as a high neutralization of phages. The *K* rate was estimated using the formula *K* = 2.3 × (*D*/*T*) × log (P0/Pt), where *K* is the rate of phage inactivation, *D* is the reciprocal of the serum dilution, *T* is the time in minutes during which the reaction occurred (30 min), P0 is the phage titer at the start of the reaction, and Pt is the phage titer at time *T*.

Statistical analysis for *K* rate was performed using the Wilcoxon rank sum test (dependent trials) or Mann-Whitney *U* test (independent trials). The level of statistical significance (*p*) was calculated for frequencies of results of PT by the *V*-squared test. *p* < 0.05 was considered as significant.

### Categories of the outcome of PT

The outcome of PT was evaluated according to Międzybrodzki et al. (2012).

Categories A–C were considered as positive responses to PT: A—pathogen eradication and/or recovery (eradication confirmed by the results of bacterial cultures; recovery refers to wound healing or complete subsidence of the infection symptoms), B—good clinical result (almost complete subsidence of some infection symptoms, together with a significant improvement of the patient’s general condition after completion of PT), C—clinical improvement (discernible reduction in the intensity of some infection symptoms after completion of PT to a degree not observed before PT, when no treatment was used).

Categories D–G were considered as inadequate responses to PT: D—questionable clinical improvement (reduction in the intensity of some infection symptoms to a degree that could also be observed before PT), E—transient clinical improvement (reduction in the intensity of some infection symptoms observed only during application of phage preparations and not after termination of PT), F—no response to treatment (lack of reduction in the intensity of some infection symptoms observed before PT), G—clinical deterioration (exacerbation of symptoms of infection at the end of PT).

## Results

AAS was examined by phage neutralization test in 25 patients with chronic rhinosinusitis who received phages locally (*n* = 4) or locally and orally (*n* = 21) (Table 1; Table 2). Twenty-three patients used *Staphylococcus aureus* phages and 3 patients used *Pseudomonas aeruginosa*, *Klebsiella pneumoniae*, or *Escherichia coli* phages. AAS control consisted of 30 sera of healthy individuals. The mean phage inactivation *K* rate in sera of healthy subjects for *S. aureus ϕ*200 phage was 0.17 ± 0.45. Two groups of patients were distinguished: (1) with a positive responses to PT (categories A–C) (*n* = 8) (Table 1) and (2) inadequate responses to PT (categories (D–G) (*n* = 17) (Table 2). Patients in group 1 prior to PT showed low mean *K* rate = 0.23 ± 0.32 and in group 2 before PT, the mean *K* rate was also low at 0.14 ± 0.17. During PT, the *K* rate increased significantly (Wilcoxon test, *p* < 0.05) in group patients 1 and 2 (mean *K* rate = 9.50 ± 16.28 (days 14 to 62) vs. mean *K* = 10.92 ± 23.43 (days 14 to 63), respectively). When the *K* rates during therapy between the group of patients with a positive responses to therapy and the group with inadequate responses to therapy were compared, no statistically significant differences were found (Mann-Whitney *U* test, *p* = 0.65) suggesting no differences between the *K* levels in both clinically analyzed groups.

Examination of the *K* rates in all patients, using phages locally or locally and orally, has revealed low phage neutralization before PT (mean *K* = 0.17 ± 0.23) (Table 3). The *K* rate increased significantly (Wilcoxon test, *p* < 0.05) in patients during PT (mean *K* = 10.41 ± 20.84). Then, 6 patients out of 25 (24%) had a high phage inactivation *K* rate (above 18) during PT (days 26 to 62). During PT, patients with *K* < 5 (*n* = 18) had a positive result of PT in 27.8% of cases (*n* = 5), while patients with *K* > 18 (*n* = 6) had a positive result of PT in 33.3% of cases (*n* = 2) suggesting that the level of phage neutralization does not affect the clinical outcome of PT. The differences were not statistically significant between the frequency of cases with positive responses to PT for patients with *K* < 5 and *K* > 18 (*V*-squared test; *p* = 0.79).

**Table 3:** Outcome of PT depending on *K* for patients with chronic rhinosinusitis using phages locally or locally and orally

Parameters	Before PT	During PT	*p* (Wilcoxon test)
Outcome of PT for patients using phages locally or locally and orally *n* = 25			
Mean *K* ± SD*	0.17 ± 0.23	10.41 ± 20.84	0.00001
Outcome of PT (A–C)^a^, *n* (%)	–	8 (32%)	–
Outcome of PT (D–G)^b^, *n* (%)	–	17 (68%)	–
Outcome of PT for patients using phages locally or locally and orally *n* = 25 depending on *K*			
	*K* < 5; *n* = 18	*K* = 5–18; *n* = 1	*K* > 18; *n* = 6
Mean *K* ± *SD* before PT	0.12 ± 0.15	0.85	0.27 ± 0.28
Mean *K* ± *SD* during PT*	0.59 ± 0.93	12.06	44.51 ± 23.30
*p* (Wilcoxon test)	0.0003	–	0.03
Outcome of PT (A–C)^a^, *n* (%)	5 (27.8%)^#^	n = 1	2 (33.3%)^#^
Outcome of PT (D–G)^b^, *n* (%)	13 (72.2%)	–	4 (66.7%)

*K* rate of phage inactivation, *PT* phage therapy, *SD* standard deviation

*K* < 5, low neutralization of phages

*K* = 5–18, medium neutralization of phages

*K* > 18, high neutralization of phages

^a^Results A–C positive responses to PT

^b^Results D–G inadequate responses to PT

*Significantly associated with the increase in the *K* rate during PT compared to the *K* rate before PT (Wilcoxon test; *p* < 0.05)

^#^There was no statistically significant difference (*V*-squared test; *p* = 0.79) in the frequencies between positive responses to PT for patients with *K* < 5 and *K* > 18

Analysis of the AAS level in the group of patients with chronic rhinosinusitis in relation to the results of PT reveals lack of significant correlation between those two parameters.

## Discussion

The data presented in this communication confirms our previous suggestions derived from studies conducted on a heterologous group of 20 patients with soft tissue infections, bone infections, and respiratory infections showing that good therapy outcome may be noted in patient groups with both low and high anti-phage responses (*K* < 5 and *K* > 18) (Łusiak-Szelachowska et al. 2017). We have suggested that local interactions between phages and antibodies capable of penetrating the sites of bacterial infections are more relevant for the outcome of PT than such interactions in the peripheral blood (Górski et al. 2019b). Antibody production to intravenously administered phage has been used in the diagnostics and monitoring in patients with immunodeficiency syndrome (Ochs et al. 1971). Thus, the appearance of anti-phage antibodies in the blood could be a marker of immune recovery during PT and constitute a good prognostic sign, at least in some patients (Górski et al. 2019b).

Evidently, more data is needed to determine the role of anti-phage antibody responses in the success of PT. Importantly, all relevant clinical trials should include that parameter to provide the necessary information.
